# The Non-Simultaneous Enhancement of Phosphorus Acquisition and Mobilization Respond to Enhanced Arbuscular Mycorrhization on Maize (*Zea mays* L.)

**DOI:** 10.3390/microorganisms7120651

**Published:** 2019-12-04

**Authors:** Junli Hu, Xiangchao Cui, Junhua Wang, Xiangui Lin

**Affiliations:** 1 State Key Laboratory of Soil and Sustainable Agriculture, Institute of Soil Science, Chinese Academy of Sciences, Nanjing 210008, China; jlhu@issas.ac.cn (J.H.); cuixih198878@163.com (X.C.); jhwang@issas.ac.cn (J.W.); 2 University of Chinese Academy of Sciences, Beijing 100049, China; 3 School of Geographic Sciences, Xinyang Normal University, Xinyang 464000, China

**Keywords:** *Funneliformis mosseae*, NPK fertilization, soil alkaline phosphatase, soil available P, soil pH, *ZEAma*;*Pht1*;*6*

## Abstract

Arbuscular mycorrhizal (AM) fungi can ameliorate not only plant phosphorus (P) nutrition but also soil P mobilization, while P mobilization occurs secondarily and may in turn limit P acquisition at certain crop growth stages. It can be termed as the “mycorrhiza-inducible P limitation”, which has so far largely escaped study. A pot experiment was conducted to test the dynamic P acquisition of maize (*Zea mays* L.) at the vegetative growth stage and P mobilization in the soil in response to AM fungal inoculation in an unsterilized arable alkaline soil. The experiment included two fertilization levels and two AM inoculation levels, i.e., nitrogen (N), P, and potassium (K) fertilization (NPK) and non-fertilization (control), as well as *Funneliformis mosseae* inoculation (+M) and non-inoculation (–M). Regardless of fertilization, +M increased mycorrhizal colonization and plant biomass at weeks 4 and 8 but increased tissue P concentration only at week 4 compared with those of –M. In addition, the plant P acquisition and shoot biomass in the control+M treatment at weeks 4 and 8 were close to and much lower than those of NPK–M, respectively. Furthermore, the increase in soil P mobilization potential, which was achieved by the accelerated soil alkaline phosphatase activity and the decreased soil pH, was lower than the increase in root P-acquiring efficiency, which was achieved by the enhanced mycorrhization and *ZEAma;Pht1;6* (a mycorrhiza- inducible Pi transporter in maize root) expression. Regardless of fertilization, +M thus significantly decreased soil available P concentrations compared with those in the –M treatments. Therefore, there was a large, real gap between soil P mobilization and root P acquisition in response to enhanced root mycorrhizal colonization, substantially limiting plant P acquisition and growth.

## 1. Introduction

As Earth’s human population steadily increases, the demand placed on the agriculture industry to supply sufficient food is becoming one of the greatest challenges facing the agrarian community. To cope with this demand, a great deal of effort in analyzing agro-ecosystems and soil biological systems as a whole is needed to better understand the interactions and complex processes governing agricultural land stability [1]. Among soil organisms, arbuscular mycorrhizal (AM) fungi are ubiquitous mutualists that form beneficial and symbiotic associations with the roots of the majority of terrestrial plant species [2,3]. The association between AM fungi and plant roots develops in two functional phases: the intraradical phase including intercellular hyphae and specialized arbuscules, and the extraradical phase with hyphae extending from root into soils [4]. AM fungi can link soil and roots directly, increasing the root’s effective absorption zone using extraradical hyphae that explore the soil away from the root surface [5]. Therefore, the most important of the symbiotic benefits is the increased nutrient uptake, notably phosphorus (P) acquisition [6].

It has been widely reported that, as one of the three major essential macronutrients, P always limits plant growth due to its low availability in soils [7] and the relatively low capture efficiency of P fertilizers by crop roots during the year of application [8]. Fortunately, P uptake through the mycorrhizal pathway usually can increase plant growth dramatically [9] and is vital for plant growth in nutrient-poor soils [10]. However, P deficiency severely limits plant growth due to the low supply capacity of soil available P rather than the mycorrhiza-mediated P uptake capability in subterranean roots, since mycorrhization is always high in such P-limited soils [11,12]. Therefore, increasing the size of the soil available P pool, a major and even the prime limiting factor for plant growth [1], is as important as improving root P acquisition capability. Fertilizers are used primarily to increase P availability to crops worldwide. However, farmers are often forced to make decisions about their fertilization strategy that reflects economic rather than agronomic pressure [13]. It has been well documented that overuse of P in agricultural settings is a key challenge to the sustainability of the finite global P resource for food production, which not only leads to the rapid exhaustion of limited P reserves but also causes the exacerbation of aquatic ecosystems as well as associated harmful consequences [14].

To obtain the benefits of AM fungi in acquiring P, the soil available P status needs to be raised to an appropriate level. Fortunately, the influences of mycorrhizal traits on plant P uptake efficiency include not only the uptake kinetics but also soil available P enhancement [15]. For example, the enhancement of phosphatase activity in soils is one of the vital roles involved in “mycorrhization effect” on soil P mobilization. Mycorrhizal roots can not only release more enzymes owing to improved nutrition and/or larger root system, but also alter soil microbial metabolic activities via changing root exudation patterns or fungal exudates [5]. As a result, soil phosphatase activity could be used as an indicator to reveal changes in soil P mobilization activity upon mycorrhization. However, it was hypothesized that the relatively delayed acceleration of soil phosphatase activity, which occurs secondarily to mycorrhization, may limit root P acquisition again at certain crop growth stages. This phenomenon can be termed as the “mycorrhiza-inducible P limitation” and has so far largely escaped study or even notice.

The direct P acquisition pathway, via root hairs and epidermal cells, and the indirect pathway, via fungal hyphae, may operate together to acquire P effectively for mycorrhizal plants [16,17]. It has been consistently demonstrated that AM fungi have a high-affinity P-uptake capability that enhances plant P nutrition, and the underlying mechanisms have been systematically discussed [17,18,19]. However, uncertainties usually remain about the direct contribution of mycorrhization to plant P uptake. Phosphate (Pi) transporters play important roles in Pi transfer for both direct and indirect P uptake pathways [20,21]. Among root Pi transporters, the mycorrhiza-inducible ones are involved in the assimilation of Pi that are released by AM fungus at the so-called symbiotic interface, and can be used as valuable markers in the root for assessing the symbiotic P uptake pathway [22,23,24]. For example, the gene *ZEAma;Pht1;6* has been identified as a mycorrhiza-inducible Pi transporter; its expression is strong and limited in mycorrhizal and non- mycorrhizal maize (*Zea mays* L.) roots, respectively [25,26]. Thus, root *ZEAma;Pht1;6* expression could be used as a direct indicator to monitor alterations in root P acquisition capability due to enhanced mycorrhization.

Experiments performed under sterilized conditions are quite different from those actually encountered in fields. In order to find a practical mycorrhizal application that can increase P use efficiency, it is better to test the effects of mycorrhizal inoculation on P acquisition and mobilization under unsterilized conditions. In a previous study, increased alkaline phosphatase (ALP) activity and available P concentration with AM fungal inoculation were obtained in a sandy loam soil after a whole maize growth period (>15 weeks) [15]. However, it was not resolved whether there was a gap between soil P mobilization and root P acquisition in response to AM fungal inoculation, or whether the gap influenced plant P acquisition during maize plant growth. Therefore, more attention should be paid to the vegetative growth stages, i.e., the early and middle stages. The objectives of the current 8-week study were therefore to investigate the changes in tissue P concentration and root *ZEAma;Pht1;6* expression in maize plants, as well as in soil available P concentration and soil ALP activity, in response to AM fungal inoculation and to discuss whether there is a gap between the enhancement of root P acquisition and soil P mobilization with enhanced mycorrhization.

## 2. Materials and Methods

### 2.1. Soil and Mycorrhizal Inoculum Preparation

A sandy loam soil sample was collected from an agricultural field in Fengqiu County (35°00′ N, 114°24’ E), Henan Province, China. The soil, which was derived from the alluvial sediments of the Yellow River, was classified as an aquic inceptisol. The air-dried soil sample was ground using a wooden pestle, and immediately homogenized with a 5 mm sieve. The soil had a pH of 8.56 (H_2_O) and contained 0.52 g/kg total P and 4.27 mg/kg available P (i.e., Olsen-P).

*Funneliformis mosseae* (Nicol. & Gerd.) Gerd. & Trappe, which could induce higher maize yields than other AM fungal species [27], is widely used in P uptake experiments for maize [28,29]. The *F. mosseae* M47V was obtained from the International Bank for Glomeromycota (Dijon, France) and was further propagated on white clover (*Trifolium repens* L.) grown in an autoclaved (121 °C for 1 h on 3 successive days) substrate for two successive propagation cycles of 4 months each. The final inocula comprised a mixture of rhizosphere soil containing hyphae, spores, and root fragments, and were air-dried and homogenized with a 2 mm sieve. The non-mycorrhizal inoculum was prepared under the same conditions with the same sterilized substratum on which white clover was cultivated.

### 2.2. Pot Experiment

There were four treatments with two fertilization levels and two AM inoculation levels, i.e., nitrogen (N), P, and potassium (K) fertilization (NPK) and non-fertilization (control), and *F. mosseae* inoculation (+M) and non-inoculation (–M). Soil samples of 2.4 kg each were put in a polyvinyl chloride pot (18 cm diameter × 18 cm depth), followed by a thin layer of 150 g AM inocula or non-mycorrhizal inoculum, five maize seeds and 0.6 kg casing soils. For the two NPK treatments, the soil samples were completely mixed with chemical fertilizers before the experiment, and the application rates of N, P_2_O_5_, and K_2_O were equal to 150, 60 and 150 kg/ha, respectively. The pots were randomly arranged with four replicates per treatment. After emergence, the maize seedlings were thinned to four per pot. Plants were grown in a sunlit greenhouse with 30/22 °C day/night temperature and 40–60% relative humidity, and the soil was maintained at 60–70% of water-holding capacity. Two maize plants per pot were carefully harvested after growing for 28 days (i.e., 4 weeks) with a little spoon, and the other two were finally harvested after growing for 56 days (i.e., 8 weeks).

### 2.3. Mycorrhizal Colonization, Plant Biomass, and P Concentration Analysis

The maize plants were divided into roots and shoots. All fresh roots harvested at week 4 and roots only from a randomly selected maize plant per pot harvested at week 8 were thoroughly rinsed with tap water and weighed before drying. Weighed subsamples of fresh roots were cleaned with 10% (*m*/*v*) KOH and stained with acid fuchsin, and the grid-line intersect method was used to assess mycorrhizal colonization [30,31]. All remaining root and shoot samples were weighed after oven drying at 70 °C for 48 h. Subsamples of dried and ground shoots and roots were taken for immediate acid digestion in HNO_3_ (70%): HClO_4_ (70%) mixture (6:1 *v*/*v*) [32], followed by molybdenum- ascorbic acid colorimetry to measure tissue P concentration [33]. In this way, the average individual P acquisition (the total amount of P accumulated in a maize plant) was calculated for each pot.

### 2.4. Root ZEAma;Pht1;6 Expression Analysis

At week 8, all fresh roots harvested from a different maize plant in each pot were quickly washed and snap-frozen in liquid N_2_ until gene expression analysis. Total RNA was extracted from approximately 80 mg maize roots using the EASYspin Plus Plant RNA Kit (Zoonbio Biotech, Nanjing, China). Approximately 0.5 μg of total RNA was used as a template for first-strand cDNA synthesis using HiScript QRT SuperMix for qPCR (+gDNA wiper) (Vazyme Biotech, Nanjing, China). Random primers supplied by the kit were used during the reverse transcription reaction. Finally, 20 μL of solution containing 10 μL 2×SYBR Green qPCR Mix (Zoonbio Biotech), 0.4 μL *ZEAma*;*Pht1*;*6* forward primer, 0.4 μL *ZEAma*;*Pht1*;*6* reverse primer, 2 μL of cDNA template and 7.2 μL double-distilled water was used for real-time PCR analysis by LightCycler 480 II (Roche, Basel, Switzerland). The thermal cycling consisted of an initial denaturation at 94 °C for 30 s, followed by 45 cycles of denaturation at 94 °C for 20 s, annealing/extension at 55 °C for 20 s, and 72 °C for 30 s. At the third step of the cycle, the fluorescence signal data were obtained to eliminate the signal of possible primer dimers. Each reaction was performed in triplicate, and the mean threshold cycle value was finally calculated for each sample. To calibrate gene expression data, *γ*-tubulin, a maize housekeeping gene, was used as a control [34]. The determination of *γ*-tubulin gene expression followed the same protocol. The forward and reverse primers were 5′-ACATAAACGCCCTCAAG GAG-3′ and 5′-GACGGTGACCCAGTAGCC-3′ for *ZEAma*;*Pht1*;*6*, and 5′-GTCCTGTGCCACTCTA TTGC-3′ and 5′-CTTGTTTCCACCTGATTTGG-3′ for *γ*-tubulin [35]. The real-time PCR data are presented as the following equation:

Relative expression = (*E*_target_)^*Δ*Ct target (control-sample)^/(*E*_reference_)^reference (control-sample)^.

### 2.5. Soil pH, ALP Activity, and Available P Concentration Analysis

After all plants were harvested at week 8, all soil samples in each pot was homogenized immediately. Subsamples (approximately 100 g) were collected, air-dried, and homogenized again with a 2 mm sieve. Soil pH was measured with a glass electrode at a soil: water ratio of 1:2.5 (*m*/*m*). Soil ALP activity was assayed using 2 g (wet weight) aliquots of soil according to [36] and is given in units of mg *p*-nitrophenol produced by 1 g soil within 24 h. Soil available P was determined by the molybdenum-blue method after extraction by sodium bicarbonate [37]. All these results were expressed on an oven-dried (105 °C, 24 h) soil weight basis.

### 2.6. Statistical Analysis

The means and standard deviations for the four replicates were calculated. The comparison of mean effects was performed with SPSS software based on the Duncan’s new multiple range method (*p* < 0.05). Redundancy analysis (RDA), a multivariate direct gradient analysis, was performed by Canoco software to elucidate the relationships between plant/mycorrhizal parameters (i.e., mycorrhizal colonization, *ZEAma*;*Pht1*;*6* expression, biomass, and P concentration and acquisition), soil properties (i.e., pH, available P concentration, and ALP activity), and different treatments. To this end, the Pearson correlation coefficients were also calculated among soil and plant/mycorrhizal parameters to assess the significance of the relationships revealed by RDA.

## 3. Results

### 3.1. Mycorrhizal Colonization, Plant Biomass and P Acquisition

**Figure 1:**
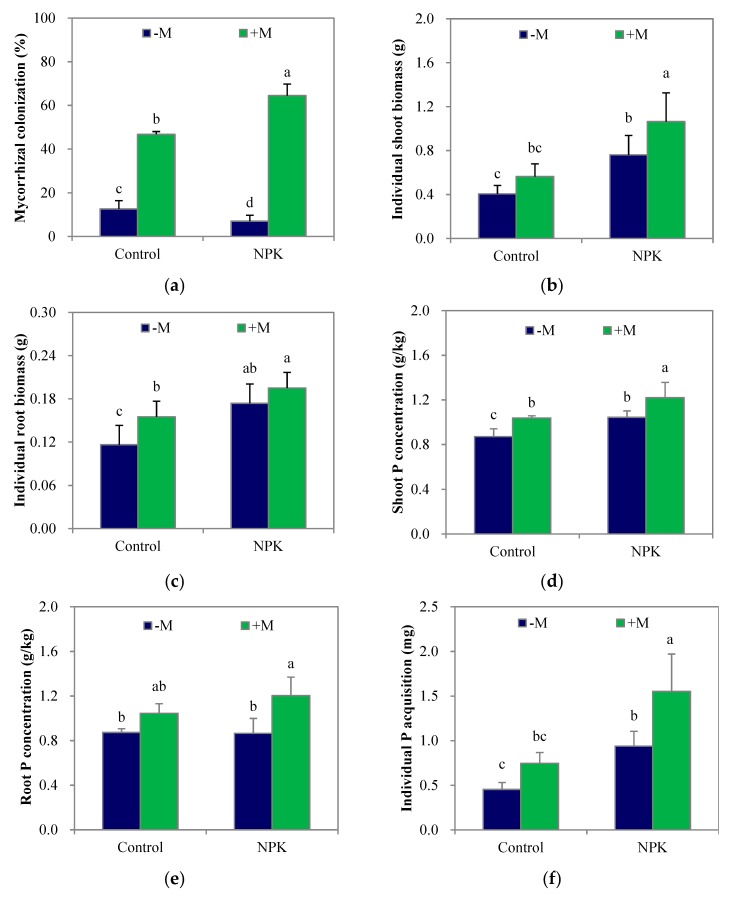
Mycorrhizal colonization (**a**), the individual biomasses and P concentrations of shoot and root (**b**–**e**), and the individual P acquisition (**f**) of maize plant after growing for 4 weeks. NPK, NPK fertilization; Control, non-fertilization; +M, *Funneliformis mosseae* inoculation; −M, non-inoculation. Vertical T bars indicate standard deviations. Values not topped by a same letter differ significantly (*p* < 0.05).

Mycorrhization appeared in all four treatments, including the two non-inoculation (–M) treatments, since indigenous AM fungi existed in the soil (Figure 1a). However, NPK fertilization significantly decreased (*p* < 0.05) the colonization by indigenous AM fungi. In contrast, fertilization significantly increased (*p* < 0.05) maize shoot and root biomass (Figure 1b; Figure 1c), shoot P concentration (Figure 1d) and individual plant P acquisition (Figure 1f) but not root P concentration (Figure 1e) compared with those of the unfertilized treatment. Regardless of fertilization, *F. mosseae* inoculation (+M) significantly increased (*p* < 0.05) mycorrhizal colonization, and tended to increase the biomass, P concentration and acquisition of maize plants compared with those in the uninoculated treatments. Specifically, +M significantly increased (*p* < 0.05) shoot P concentration and root biomass in the control soil and shoot biomass, shoot and root P concentrations, and individual plant P acquisition in the NPK soil. In addition, there were no significant differences between the control+M and NPK–M treatments, except in the root mycorrhizal colonization rate.

**Figure 2:**
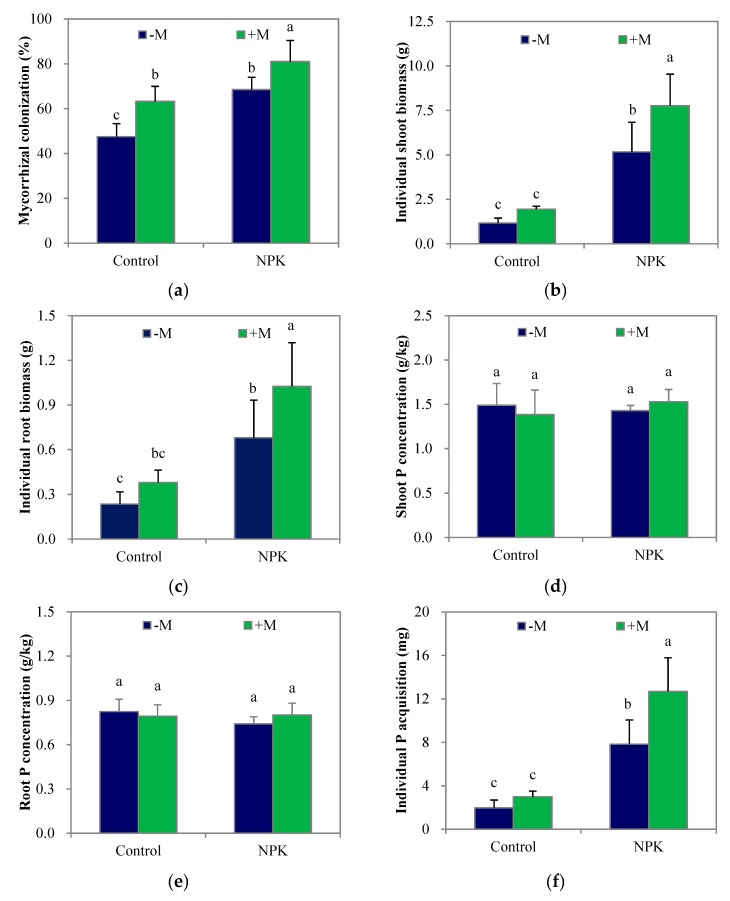
Mycorrhizal colonization (**a**), the individual biomasses and P concentrations of shoot and root (**b**–**e**), and the individual P acquisition (**f**) of maize plant after growing for 8 weeks. NPK, NPK fertilization; Control, non-fertilization; +M, *Funneliformis mosseae* inoculation; –M, non-inoculation. *Vertical T bars* indicate standard deviations. Values not topped by a same letter differ significantly (*p* < 0.05).

At week 8, the average mycorrhizal colonization rates were substantially higher than those at week 4 (Figure 2a). The adverse effect of fertilization on indigenous AM fungi was no longer apparent, and fertilization significantly increased (*p* < 0.05) maize shoot and root biomass (Figure 2b; Figure 2c) and individual plant P acquisition (Figure 2f) over those of the unfertilized treatment as before, but had no significant effect on shoot or root P concentration (Figure 2d; Figure 2e).

Compared with the data obtained at week 4, the average shoot P concentrations in the control–M and NPK–M treatments increased by 71% and 37%, respectively, while the average root P concentrations decreased by 6% and 14%, respectively. On the other hand, +M significantly increased (*p* < 0.05) root mycorrhizal colonization regardless of the fertilization treatment, but the enhancement in P concentration in either shoots or roots that were obtained at week 4 had all disappeared by week 8. The average shoot P concentrations in the control+M and NPK+M treatments increased only by 33% and 25%, respectively, while the average root P concentrations decreased by 24% and 33%, respectively. Furthermore, +M significantly increased (*p* < 0.05) both shoot and root biomass and individual plant P acquisition in the NPK soil but tended to increase maize root biomass only in the control soil. In addition, the individual plant P acquisition and shoot biomass in the control+M treatment were much lower (*p* < 0.05) than those of NPK–M, respectively.

### 3.2. Mycorrhiza-Inducible Pi Transporter Expression and Soil P Mobilization

**Figure 3:**
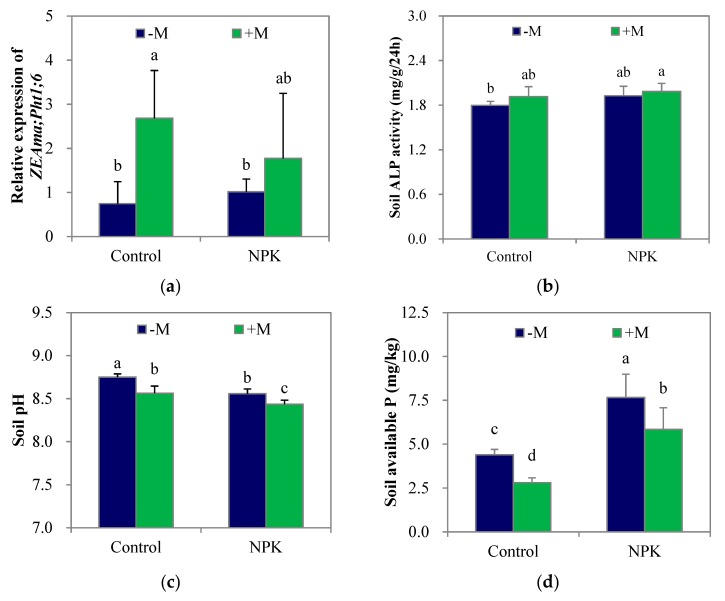
The relative expression of *ZEAma*;*Pht1*;*6* in maize root (**a**), soil alkaline phosphatase (ALP) activity (**b**), soil pH (**c**), and soil available P concentration (**d**) after growing for 8 weeks. NPK, NPK fertilization; Control, non-fertilization; +M, *Funneliformis mosseae* inoculation; −M, non-inoculation. Vertical T bars indicate standard deviations. Values not topped by a same letter differ significantly (*p* < 0.05).

At week 8, the control–M soil had an available P concentration of 4.39 mg/kg, which was equal to the original level. Compared with the unfertilized treatments, NPK fertilization significantly increased (*p* < 0.05) soil available P concentration (Figure 3d) and tended to increase soil ALP activity (Figure 3b), while significantly decreasing (*p* < 0.05) soil pH (Figure 3c). Nevertheless, NPK fertilization had no significant effect on the relative expression of *ZEAma*;*Pht1*;*6*, a mycorrhiza- inducible Pi transporter, in the maize root (Figure 3a). On the other hand, +M significantly increased (*p* < 0.05) the relative expression of *ZEAma*;*Pht1*;*6* in maize roots in the control soil rather than in the NPK soil. Regardless of fertilization, +M tended to increase soil ALP activity but significantly decreased (*p* < 0.05) soil pH and soil available P concentrations compared with those in the –M treatments.

### 3.3. RDA Results

**Figure 4:**
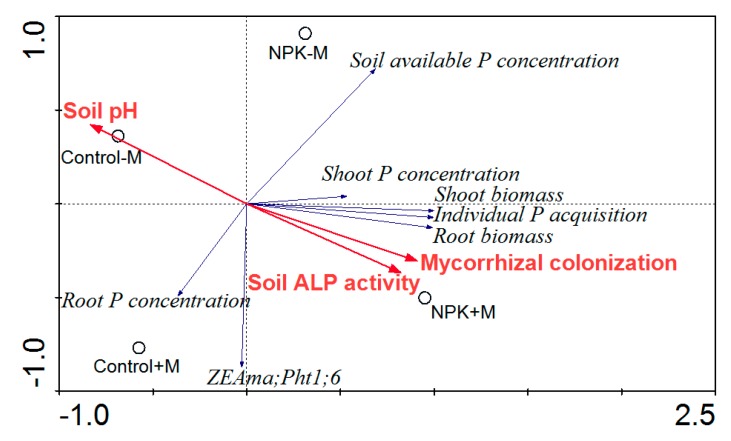
Redundancy analysis (RDA) plot of plant/mycorrhizal parameters with soil properties as affected by different treatments. NPK, NPK fertilization; Control, non-fertilization; +M, *Funneliformis mosseae* inoculation; −M, non-inoculation; ALP, alkaline phosphatase.

In the RDA ordination plot (Figure 4), compared with control–M, both control+M and NPK–M had decreasing effects on soil pH and positive effects on mycorrhizal colonization and soil ALP activity, while NPK+M had the greatest influences on those variables. The soil available P concentration varied in the following order: control+M < control–M < NPK+M < NPK–M. The shoot biomass was significantly correlated (*p* < 0.01) with root biomass, and were significantly correlated (*p* < 0.05; *p* < 0.01) with mycorrhizal colonization (Table 1). The individual P acquisition was significantly correlated (*p* < 0.01) with both shoot and root biomass, and both the individual P acquisition and soil ALP activity were significantly correlated (*p* < 0.05; *p* < 0.01) with mycorrhizal colonization, while soil pH was negatively but closely correlated (*p* < 0.01) with both mycorrhizal colonization and soil ALP activity.

## 4. Discussion

The objectives of this study were to test whether there was a gap between soil P mobilization and root P acquisition in response to AM fungal inoculation and enhanced mycorrhization, and to examine whether the gap influenced plant P acquisition at the vegetative growth stage of maize. After growing for 4 weeks, the colonization by indigenous AM fungi was adversely influenced by the greatly increased amounts of readily soluble P in the NPK-fertilized soil, while the enhanced mycorrhizal colonization with *Funneliformis mosseae* inoculation induced higher P concentrations in both shoots and roots, notably with the NPK-fertilized maize (Figure 1d,e). The colonization by *F. mosseae* was not negatively influenced by fertilization since the AM inocula were inoculated as a thin layer around maize seeds rather than being completely mixed in the fertilized soils. As a result, the amounts of readily soluble P around the inoculated AM fungi were much lower than those around the indigenous AM fungi. At this point, there were no perceptible P limitations at the seedling stage of maize. It is noteworthy that the increase in P acquisition with *F. mosseae* inoculation was significant in the NPK soil rather than in the control soil (Figure 1f), suggesting that the exploitation of the enhanced root P acquisition capability was relatively limited by the low supply capacity of soil available P.

After growing for 8 weeks, the adverse effect of fertilization on indigenous AM fungi was no longer apparent, suggesting a decrease in the amount of easily soluble P in the soil as the plant grew and/or an increased dependency on mycorrhiza for plant growth relative to that of the maize plants grown in the control soil. Regardless of NPK fertilization, the increases (25–33%) in shoot P concentration and the decreases (3–24%) in root P concentration in the fungi-inoculated maize from week 4 to week 8 were much lower and higher, respectively, than those (37–71% and 6–14%) of the non-inoculated maize. As a result, the enhancement in tissue P concentration that were obtained at week 4 all disappeared by week 8 (Figure 2d,e). However, the elevations of both mycorrhizal colonization and *ZEAma*;*Pht1*;*6* expression were continuous (Figure 2a; Figure 3a), suggesting that the enhanced root P acquisition capacity did not disappear. The transfer of inorganic Pi via the transporter *ZEAma*;*Pht1*;*6* not only supplies nutrients to the root cells but also plays a role in the maintenance of arbuscules [38].

**Figure 5:**
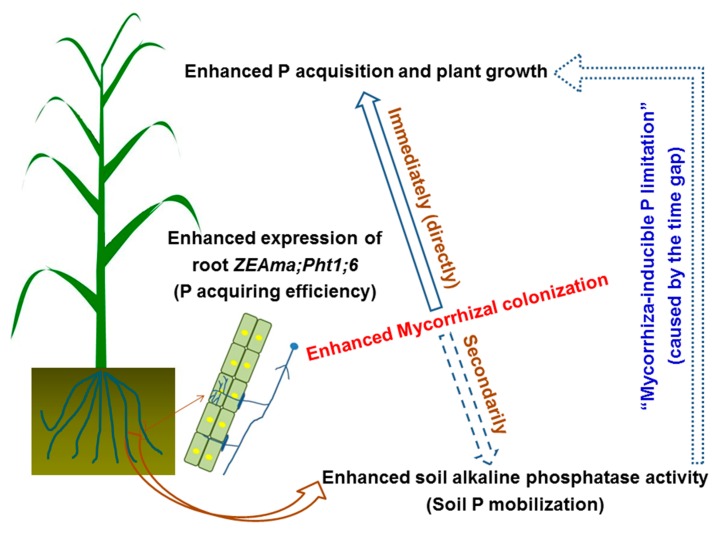
The diagrammatic sketch of “mycorrhiza-inducible P limitation” in the soil-maize system.

On the other hand, soil ALP activity also tended to increase with AM fungal inoculation (Figure 3b; Figure 4), but soil available P concentrations significantly decreased due to the greatly elevated amount of P acquired by plants (Figure 3d; Figure 4), suggesting that there was a large, real gap between soil P mobilization and root P acquisition in response to the enhanced mycorrhizal colonization at the jointing stage of maize (Figure 5), and this gap did limit plant P acquisition and growth substantially. For example, both the individual plant P acquisition and the shoot biomass of the control+M treatment were close to those of the NPK–M treatment at week 4 but were greatly lower than those of the NPK–M treatment at week 8. As a result, in addition to the lost enhancements in tissue P concentrations, the significant decreases in available P concentration in both control and NPK soils with AM fungal inoculation seem to be crucial evidence in support of the “mycorrhiza-inducible P limitation” hypothesis. It is necessary to specify that the tested pot conditions were different from the field conditions. In conditions with other P application levels, different results may arise [15,39,40], and the gap observed between increased P acquisition and insufficient P mobilization may diminish.

In addition to soil phosphatase activity, soil pH also plays an important role in determining P availability in soils. An effective mechanism by which mycorrhizal roots increase soil P availability in neutral or alkaline soils is decreasing soil pH by the production of H^+^ or by the exudation of organic acids [41]. In this experiment, regardless of NPK fertilization, AM fungal inoculation also induced a lower soil pH compared with that in the non-inoculated treatment (Figure 3c; Figure 4). However, since the mobilization potentials of the soil total P pools with the decreased soil pH and the accelerated soil ALP activity were still not significantly enhanced during this short experimental period, the apparent values of the available P concentrations in both control and NPK soils were markedly decreased due to the increased P acquisition efficiency of the plants.

Although applying P fertilizer is one of the most effective ways to increase plant growth, high application levels may increase the risk of P accumulation in soils. Therefore, improving P availability is an alternative for enhancing the efficiency of P fertilizers [32]. Since plant growth responsiveness may correlate with soils, plants, AM isotypes, and other soil abiotic or biotic factors [42,43,44], optimal conditions under which AM fungi can play the greatest role must be considered to address the gap between soil P mobilization and root P acquisition in response to enhanced mycorrhization. For example, different AM fungal species display functional diversity in Pi uptake by maize and variable effects on the expression of *ZEAma:Pht1;6*, the indicator of AM function in maize [35]. Furthermore, additional studies, notably field ones which are greatly different from pot conditions, also need to explore the combined effects of AM fungi with other beneficial microbes, such as organic P-mineralizing and inorganic P-solubilizing bacteria which play important roles in soil P mobilization [11], and other mycorrhiza-helping bacteria that promote the activity and development of AM fungi [45].

## 5. Conclusions

At week 4, regardless of NPK fertilization, AM fungal inoculation (+M) increased root mycorrhizal colonization and the biomass, P concentration, and individual P acquisition of maize plants, and the positive effects of +M on plant P acquisition and growth were close to NPK fertilization. At week 8, however, the enhancements in tissue P concentrations in +M all disappeared, and the acceleration of +M on plant P acquisition and growth were much lower than fertilization, while the elevation of mycorrhizal colonization and *ZEAma*;*Pht1*;*6* expression in +M were consistent. Since the increases in P mobilization potentials with the accelerated soil ALP activity and the decreased soil pH were lower than the increases in root P-acquiring efficiency due to the enhanced mycorrhization, the soil available P concentration significantly decreased. These results suggest a gap between soil P mobilization and root P acquisition in response to enhanced root mycorrhizal colonization, limiting plant P acquisition and growth substantially. Nevertheless, in the future, field studies (notably with different P application levels) are needed to test the “mycorrhiza-inducible P limitation” hypothesis.

## Figures and Tables

**Table 1:** The Pearson correlation coefficients between plant/mycorrhizal and soil parameters.

	Mycorrhizal Colonization	Soil pH	Soil ALP Activity	Soil Available P Concentration	Root *ZEAma*;*Pht1*;*6*	Shoot Biomass	Root Biomass
Soil pH	−0.988 **						
Soil ALP activity	0.983 **	−0.996 **					
Soil available P concentration	0.431	−0.302	0.340				
Root *ZEAma*;*Pht1*;*6*	0.381	−0.521	0.513	−0.600			
Shoot biomass	0.928 *	−0.858	0.847	0.661	0.021		
Root biomass	0.952 **	−0.895	0.880	0.593	0.106	0.996 **	
Individual P acquisition	0.922 *	−0.853	0.836	0.630	0.026	0.998 **	0.996 **

ALP, alkaline phosphatase; * *p* = 0.05, ** *p* = 0.01 (significant).

## References

[1] Khan M.S., Zaidi A., Wani P.A. (2007). Role of phosphate-solubilizing microorganisms in sustainable agriculture—A review. Agron. Sustain. Dev..

[2] Smith S.E., Read D.J. (2008). Mycorrhizal Symbiosis.

[3] Wang F., Sun Y., Shi Z. (2019). Arbuscular mycorrhiza enhances biomass production and salt tolerance of sweet sorghum. Microorganisms.

[4] Baslam M., Garmendia I., Goicoechea N. (2011). Arbuscular mycorrhizal fungi (AMF) improved growth and nutritional quality of greenhouse-grown lettuce. J. Agric. Food Chem..

[5] Wang F.Y., Lin X.G., Yin R., Wu L.H. (2006). Effects of arbuscular mycorrhizal inoculation on the growth of *Elsholtzia splendens* and *Zea mays* and the activities of phosphatase and urease in a multi-metal- contaminated soil under unsterilized conditions. Appl. Soil Ecol..

[6] Brundrett M.C. (2009). Mycorrhizal associations and other means of nutrition of vascular plants: Understanding the global diversity of hostplants by resolving conflicting information and developing reliable means of diagnosis. Plant Soil.

[7] Feng K., Lu H.M., Sheng H.J., Wang X.L., Mao J. (2004). Effect of organic ligands on biological availability of inorganic phosphorus in soils. Pedosphere.

[8] Doolette A., Armstrong R., Tang C., Guppy C., Mason S., McNeill A. (2019). Phosphorus uptake benefit for wheat following legume break crops in semi-arid Australian farming systems. Nutr. Cycl. Agroecosyst..

[9] Zheng C., Zhang J., Li X. (2013). Phosphorus supply level affects the regulation of phosphorus uptake by different arbuscular mycorrhizal fungal species in a highly P-efficient backcross maize line. Crop Past Sci..

[10] Willmann M., Gerlach N., Buer B., Polatajko A., Nagy R., Koebke E., Jansa J., Flisch R., Bucher M. (2013). Mycorrhizal phosphate uptake pathway in maize: Vital for growth and cob development on nutrient poor agricultural and greenhouse soils. Front. Plant Sci..

[11] Hu J., Lin X., Wang J., Chu H., Yin R., Zhang J. (2009). Population size and specific potential of P-mineralizing and -solubilizing bacteria under long-term P-deficiency fertilization in a sandy loam soil. Pedobiologia.

[12] Jone E.J. (2000). The effect of long-term fertilization with organic or inorganic fertilizers on mycorrhiza-mediated phosphorus uptake in subterranean clover. Biol. Fertil. Soils.

[13] Chu H., Lin X., Fujii T., Morimoto S., Yagi K., Hu J., Zhang J. (2007). Soil microbial biomass, dehydrogenase activity, bacterial community structure in response to long-term fertilizer management. Soil Biol. Biochem..

[14] Chowdhury R.B., Milne N., Chakraborty P., Ferranti P., Berry E.M., Anderson I.R. (2019). Overuse of phosphorus resources. Encyclopedia of Food Security and Sustainability.

[15] Hu J., Lin X., Wang J., Dai J., Cui X., Chen R., Zhang J. (2009). Arbuscular mycorrhizal fungus enhances crop yield and P-uptake of maize (*Zea mays* L.): A field case study on a sandy loam soil as affected by long-term P-deficiency fertilization. Soil Biol. Biochem..

[16] Bucher M. (2007). Functional biology of plant phosphate uptake at root and mycorrhiza interfaces. New Phytol..

[17] Smith S.E., Jakobsen I., Grønlund M., Smith F.A. (2011). Roles of arbuscular mycorrhizas in plant phosphorus nutrition: Interactions between pathways of phosphorus uptake in arbuscular mycorrhizal roots have important implications for understanding and manipulating plant phosphorus acquisition. Plant Physiol..

[18] Smith S.E., Smith F.A. (2011). Roles of arbuscular mycorrhizas in plant nutrition and growth: New paradigms from cellular to ecosystem scales. Annu. Rev. Plant Biol..

[19] Smith S.E., Smith F.A. (2012). Fresh perspectives on the roles of arbuscular mycorrhizal fungi in plant nutrition and growth. Mycologia.

[20] Benedetto A., Magurno F., Bonfante P., Lanfranco L. (2005). Expression profiles of a phosphate transporter gene (*GmosPT*) from the endomycorrhizal fungus *Glomus mosseae*. Mycorrhiza.

[21] Javot H., Pumplin N., Harrison M.J. (2007). Phosphate in the arbuscular mycorrhizal symbiosis: Transport properties and regulatory roles. Plant Cell Environ..

[22] Grace E.J., Cotsaftis O., Tester M., Smith F.A., Smith S.E. (2009). Arbuscular mycorrhizal inhibition of growth in barley cannot be attributed to extent of colonization, fungal phosphorus uptake or effects on expression of plant phosphate transporter genes. New Phytol..

[23] Liu F., Xu Y., Han G., Wang W., Li X., Cheng B. (2018). Identification and functional characterization of a maize phosphate transporter induced by mycorrhiza formation. Plant Cell Physiol..

[24] Plassard C., Becquer A., Garcia K. (2019). Phosphorus transport in mycorrhiza: How far are we?. Trends Plant Sci..

[25] Glassop D., Smith S.E., Smith F.W. (2005). Cereal phosphate transporters associated with the mycorrhizal pathway of phosphate uptake into roots. Planta.

[26] Nagy R., Vasconcelos M.J.V., Zhao S., McElver J., Bruce W., Amrhein N., Raghothama K.G., Bucher M. (2006). Differential regulation of five *Pht1* phosphate transporters from maize (*Zea mays* L.). Plant Biol..

[27] Chen B., Li X., Christie P. (2002). Two arbuscular mycorrhizal fungi colonizing maize under different phosphorus regimes in a compartment cultivation system. Pedosphere.

[28] Cao J., Huang Y., Wang C. (2015). Rhizosphere interactions between earthworms (*Eisenia fetida*) and arbuscular mycorrhizal fungus (*Funneliformis mosseae*) promote utilization efficiency of phytate phosphorus in maize. Appl. Soil Ecol..

[29] Watts-Williams S.J., Smith F.A., Jakobsen I. (2019). Soil phosphorus availability is a driver of the responses of maize (*Zea mays*) to elevated CO_2_ concentration and arbuscular mycorrhizal colonisation. Symbiosis.

[30] Phillips J.M., Hayman D.S. (1970). Improved procedures for clearing roots and staining parasitic and vesicular- arbuscular mycorrhizal fungi for rapid assessment of infection. Trans. Br. Mycol. Soc..

[31] Giovannetti M., Mosse B. (1980). An evaluation of techniques for measuring vesicular–arbuscular mycorrhizal infection in roots. New Phytol..

[32] Zhu Y., Smith F.A., Smith S.E. (2003). Phosphorus efficiencies and responses of barley (*Hordeum vulgare* L.) to arbuscular mycorrhizal fungi grown in highly calcareous soil. Mycorrhiza.

[33] Hanson W.C. (1950). The photometric determination of phosphorus in fertilisers using the phosphovanado–molybdate complex. J. Sci. Food Agric..

[34] Ding D., Zhang L.F., Wang H., Liu Z.J., Zhang Z.X., Zheng Y.L. (2009). Differential expression of miRNAs in response to salt stress in maize roots. Ann. Bot..

[35] Tian H., Drijber R.A., Li X., Miller D.N., Wienhold B.J. (2013). Arbuscular mycorrhizal fungi differ in their ability to regulate the expression of phosphate transporters in maize (*Zea mays* L.). Mycorrhiza.

[36] Tabatabai M.A., Page A.L., Miller R.H., Keeney D.R. (1982). Soil enzymes. Methods of Soil Analyses, Part 2, Chemical and Microbiological Properties.

[37] Olsen S.R., Cole C.V., Watanabe F.S., Dean L.A. (1954). Estimation of Available Phosphorus in Soils by Extraction with Sodium Bicarbonate.

[38] Igiehon N.O., Babalola O.O. (2017). Biofertilizers and sustainable agriculture: Exploring arbuscular mycorrhizal fungi. Appl. Microbiol. Biotechnol..

[39] Deng Y., Feng G., Chen X.P., Zou C.Q. (2017). Arbuscular mycorrhizal fungal colonization is considerable at optimal Olsen-P levels for maximized yields in an intensive wheat-maize cropping system. Field Crop Res..

[40] Duffková R., Fučík P., Jurkovská L., Janoušková M. (2019). Experimental evaluation of the potential of arbuscular mycorrhiza to modify nutrient leaching in three arable soils located on one slope. Appl. Soil Ecol..

[41] Li X.L., George E., Marschner H. (1991). Extension of the phosphorus depletion zone in VA-mycorrhizal white clover in a calcareous soil. Plant Soil.

[42] Hu J., Lin X., Wang J., Cui X., Dai J., Chu H., Zhang J. (2010). Arbuscular mycorrhizal fungus enhances P-acquisition of wheat (*Triticum aestivum* L.) in a sandy loam soil with long-term inorganic fertilization regime. Appl. Microbiol. Biotechnol..

[43] Shi S., Chen K., Gao Y., Liu B., Yang X., Huang X., Liu G., Zhu L., He X. (2016). Arbuscular mycorrhizal fungus species dependency governs better plant physiological characteristics and leaf quality of mulberry (*Morus alba* L.) seedlings. Front. Microbiol..

[44] Chen S., Zhao H., Zou C., Li Y., Chen Y., Wang Z., Jiang Y., Liu A., Zhao P., Wang M. (2017). Combined inoculation with multiple arbuscular mycorrhizal fungi improves growth, nutrient uptake and photosynthesis in cucumber seedlings. Front. Microbiol..

[45] Miransari M. (2011). Interactions between arbuscular mycorrhizal fungi and soil bacteria. Appl. Microbiol. Biotechnol..

